# A resected case of pancreatic head cancer developing 40 years after lateral pancreaticojejunostomy for chronic pancreatitis

**DOI:** 10.1007/s12328-024-01924-z

**Published:** 2024-02-24

**Authors:** Takatsugu Matsumoto, Genki Tanaka, Shozo Mori, Maiko Niki, Shun Sato, Takayuki Shiraki, Yukihiro Iso, Kazunori Nagashima, Atsushi Irisawa, Yumi Nozawa, Atsuko Takada-Owada, Kazuyuki Ishida, Taku Aoki

**Affiliations:** 1 Department of Hepato-Biliary-Pancreatic Surgery, Dokkyo Medical University, 880 Kitakobayashi, Mibu, Tochigi 321-0293 Japan; 2 Department of Gastroenterology, Dokkyo Medical University, Mibu, Tochigi Japan; 3 Department of Diagnostic Pathology, Dokkyo Medical University, Mibu, Tochigi Japan

**Keywords:** Pancreaticojejunostomy, Pancreatic head carcinoma, Resectable pancreatic cancer

## Abstract

A 72-year-old male patient presented to our department complaining of with upper abdominal pain and jaundice. He had a history of a side-to-side pancreaticojejunostomy performed 40 years previously for chronic pancreatitis. A diagnostic workup revealed a tumor 3 cm in size in the pancreatic head as the etiology of the jaundice. Subsequently, the patient was diagnosed with resectable pancreatic cancer. Following two cycles of neoadjuvant chemotherapy, an extended pancreatoduodenectomy was performed because of tumor invasion at the previous pancreaticojejunostomy site. Concurrent portal vein resection and reconstruction were performed. Pathological examination confirmed invasive ductal carcinoma (T2N1M0, Stage IIB). This case highlights the clinical challenges in pancreatic head carcinoma following a side-to-side pancreaticojejunostomy. Although pancreaticojejunostomy is believed to reduce the risk of pancreatic cancer in patients with chronic pancreatitis, clinicians should be aware that, even after this surgery, there is still a chance of developing pancreatic cancer during long-term follow-up.

## Introduction

Pancreatic cancer (PC) is the fourth leading cause of cancer-related death in the United States, with an overall survival rate of only 6%. Moreover, it is projected to become the country’s second-leading cause of cancer-related deaths by 2030 [1]. Surgical resection is currently the only curative option; however, only 20% of PCs are surgically resectable at diagnosis [2]. Chronic pancreatitis (CP), which is characterized by long-standing pancreatic inflammation, has been identified as an important risk factor for the development of pancreatic cancer [3]. The incidence of PC in patients with CP has been reported to be as high as 7.6‒68.1 times [4]. The risk has been reported with varying results across different study populations. Cumulative incidences of PC among newly diagnosed CP patients have been reported to range from 0.24 to 5.7% [4–7]. In a long-term prospective observational study of patients with chronic pancreatitis conducted by Malka et al., the incidence of pancreatic cancer in patients with chronic pancreatitis was 1.1%, with a remarkably high age- and sex-standardized incidence ratio of 26.7 times that of the National Cancer Register in France [4]. Surgical interventions for chronic pancreatitis, particularly pancreaticojejunostomy, are recognized for mitigating chronic pain and potentially reducing the long-term pancreatic cancer risk by alleviating chronic inflammation [8, 9]. Herein, we present an unusual case of pancreatic cancer that developed 40 years after a side-to-side pancreaticojejunostomy for chronic pancreatitis.

## Case presentation

A 72-year-old male patient presenting with upper abdominal pain and jaundice was referred to our department for further review. Four decades earlier, the patient underwent a side-to-side pancreaticojejunostomy, popularly recognized as the Partington procedure, for chronic pancreatitis and pancreatolithiasis. The patient also underwent open cholecystectomy for cholelithiasis. He was a heavy alcohol consumer before treatment for chronic pancreatitis but has since only consumed alcohol occasionally. Laboratory data revealed markedly elevated alkaline phosphatase and total bilirubin levels. The carbohydrate antigen 19-9 was significantly elevated (2291 U/ml) (Table 1).

**Table 1:** Laboratory data on admission

AST (U/l)	34	WBC (/mm^3^)	6600
ALT (U/l)	*53*	Hb (g/dl)	13.6
ALP (U/l)	*460*	Plt (10^4^/μl)	43.3
LDH (U/l)	*247*	PT (%)	83
T-Bil (mg/dl)	*16.8*		
D-Bil (mg/dl)	*12.2*		
TP (g/dl)	7.6		
Alb (g/dl)	4.1	CRP (mg/dl)	*0.43*
Na (mEq/l)	139		
K (mEq/l)	3.3		
Cl (mEq/l)	100	CEA (ng/ml)	4.58
UN (mg/dl)	20.4	CA19-9 (U/ml)	*2291*
Cre (mg/dl)	0.81	DUPAN-2 (U/ml)	*270*
HbA1c (%)	5.5	Elastase-1	80
		SPAN-1 (U/ml)	*120*

*Alb* albumin, *ALP* alkaline phosphatase, *ALT* alanine aminotransferase, *AFP* alpha-fetoprotein, *AST* aspartate aminotransferase, *UN* blood urea nitrogen, *CEA* carcinoembryonic antigen, *CA19-9* carbohydrate antigen 19-9, *CRP* C-reactive protein, *Cre* creatinine, *D-bil* direct bilirubin, *DUPAN-2* Duke pancreatic monoclonal antigen type 2, *HbA1c* glycated hemoglobin, *Hb* hemoglobin, *LDH* lactate dehydrogenase, *Plt* platelet, *PT* prothrombin time, *SPAN-1* S-pancreas-1 antigen, *T-bil* total bilirubin, *TP* total protein, *WBC* white blood cell

Italics show abnormal data

**Fig. 1:**
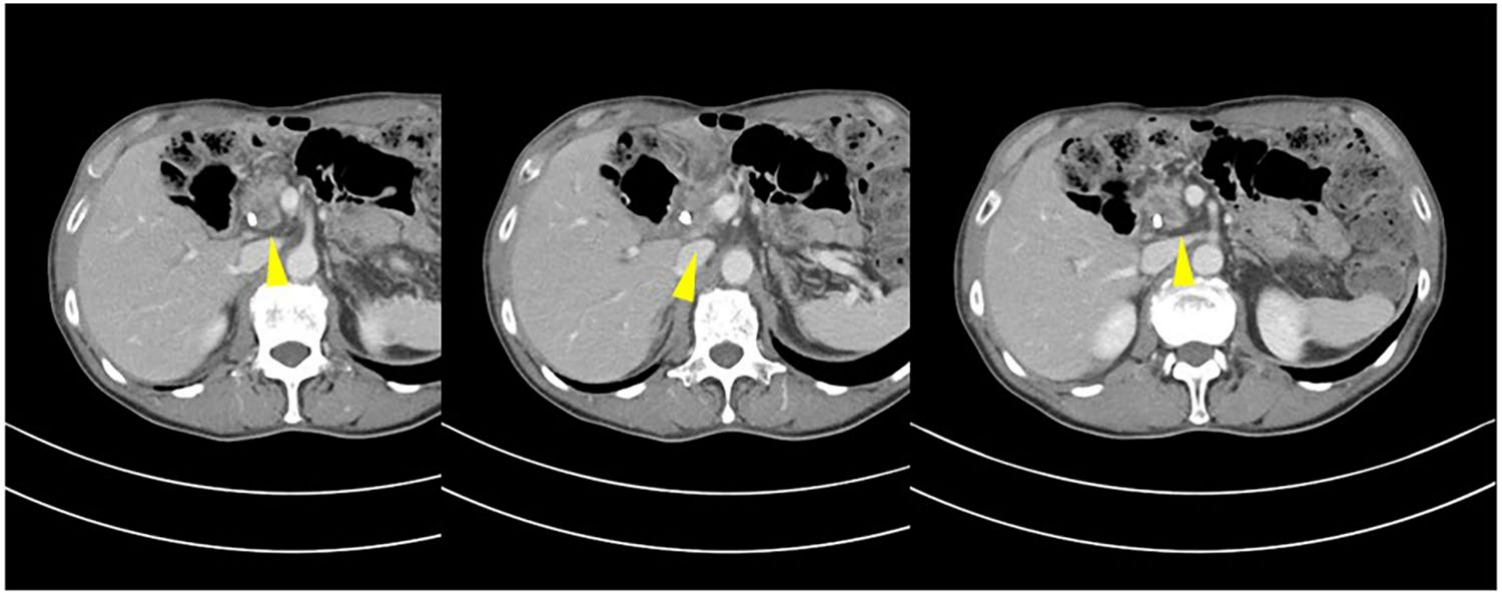
Abdominal contrast-enhanced computed tomography (portal phase). A hypoattenuated tumor with indistinct borders is visible in the pancreatic head. The tumor is in contact with the portal vein at < 180 degrees without vein contour irregularities. Arrowheads indicate pancreatic head tumor

**Fig. 2:**
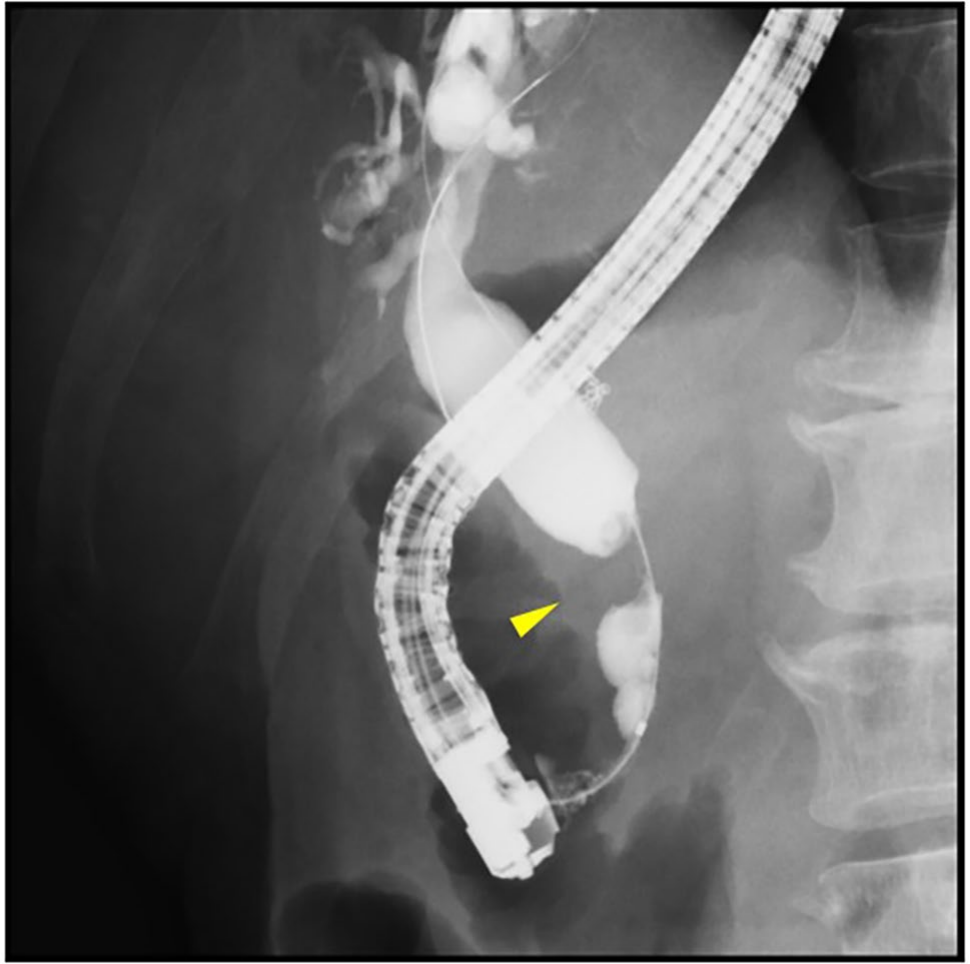
Endoscopic retrograde cholangiopancreatography. A stricture within the distal bile duct is observable. The arrowhead indicates the portion with the stricture

Contrast-enhanced computed tomography revealed a hypo-attenuated tumor located in the pancreatic head without any apparent arterial attachment. The tumor was in contact with the portal vein at < 180 degrees without vein contour irregularities (Fig. 1). Marked dilation of the intrahepatic bile duct was also observed. On the other hand, it did not show any calcification or dilation of the pancreatic duct in the pancreas. Endoscopic retrograde cholangiopancreatography revealed a stricture within the distal bile duct (Fig. 2) and hypoechoic mass 3 cm in size in the pancreatic head; sequential biopsy revealed pancreatic ductal adenocarcinoma. Positron emission tomography revealed fluorodeoxyglucose accumulation within the primary lesion (maximum standardized uptake value: 7.73); however, no signs of distant metastases were observed.

**Fig. 3:**
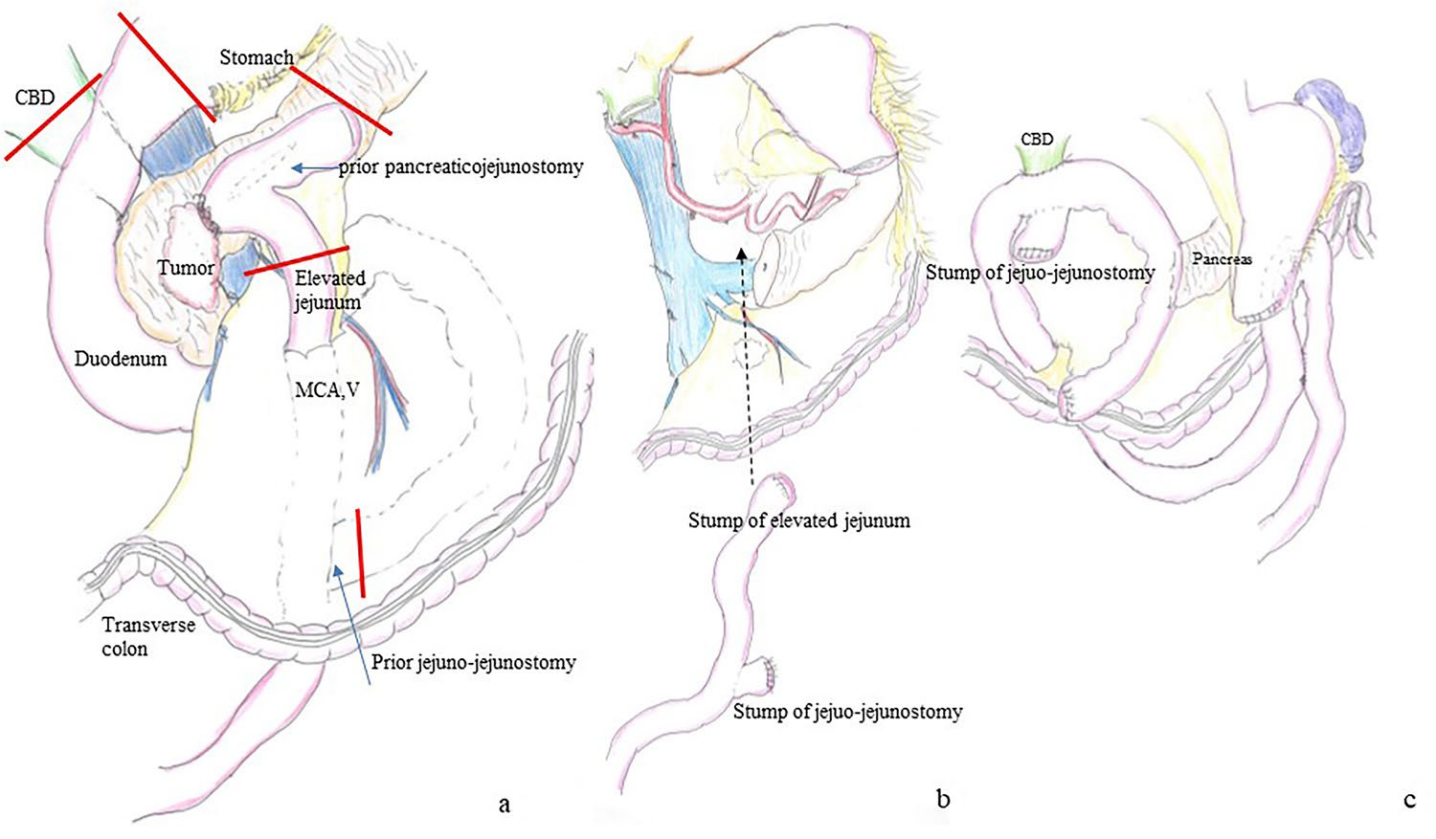
Schema of the surgery. **a** Red line indicates the cut line of each organ. Subtotal stomach was preserved. The proximal jejunum was resected to ensure adequate lymph node dissection. The jejunojejunal anastomosis was left intact to minimize the extent of jejunal resection. **b** Remnant jejunum was elevated via the retro colic route. **c** Reconstruction was performed using the modified Child method

Based on these findings, the patient was diagnosed with resectable adenocarcinoma of the pancreatic head. In accordance with the clinical practice guidelines for pancreatic cancer 2022 by the Japan Pancreas Society, he received two courses of gemcitabine and S-1 combination therapy as neoadjuvant chemotherapy (NAC). Specifically, gemcitabine was administered at a dose of 1000 mg/m^2^ on days 1 and 8, and S-1 was given orally at a dose of 80 mg/m^2^ per day from days 1 to 14, followed by a one-week rest. After completing the NAC, there was a significant decrease in the level of CA19-9 from 2291 to 151 U/ml. In addition, contrast-enhanced CT imaging showed a reduction in the tumor size from 28 to 23 mm. Additionally, positron emission tomography scans also indicated a reduction in the tumor's maximum standardized uptake value from 7.73 to 5.57. Then he underwent subsequent surgical resection. Because the tumor directly invaded the anastomosis of pancreaticojejunostomy at the previous surgery, a pancreaticoduodenectomy was performed with excision of the previous anastomosis. In addition, concurrent portal vein resection was performed, as it seemed to be invaded by the tumor. The intraoperative histological diagnosis showed that the pancreatic cut margin was negative for cancer, and the pancreatic tail was preserved. The proximal jejunum, approximately 20 cm distal to the ligament of Treitz, was resected to ensure thorough regional lymph node dissection around the superior mesenteric artery. In contrast, the jejuno-jejuno anastomosis was left intact to minimize the extent of jejunal resection, The modified Child method was used for reconstruction (Fig. 3). A drainage tube was inserted to the elevated jejunum for decompression. Operative time was 575 min, with a blood loss of 2234 ml.

**Fig. 4:**
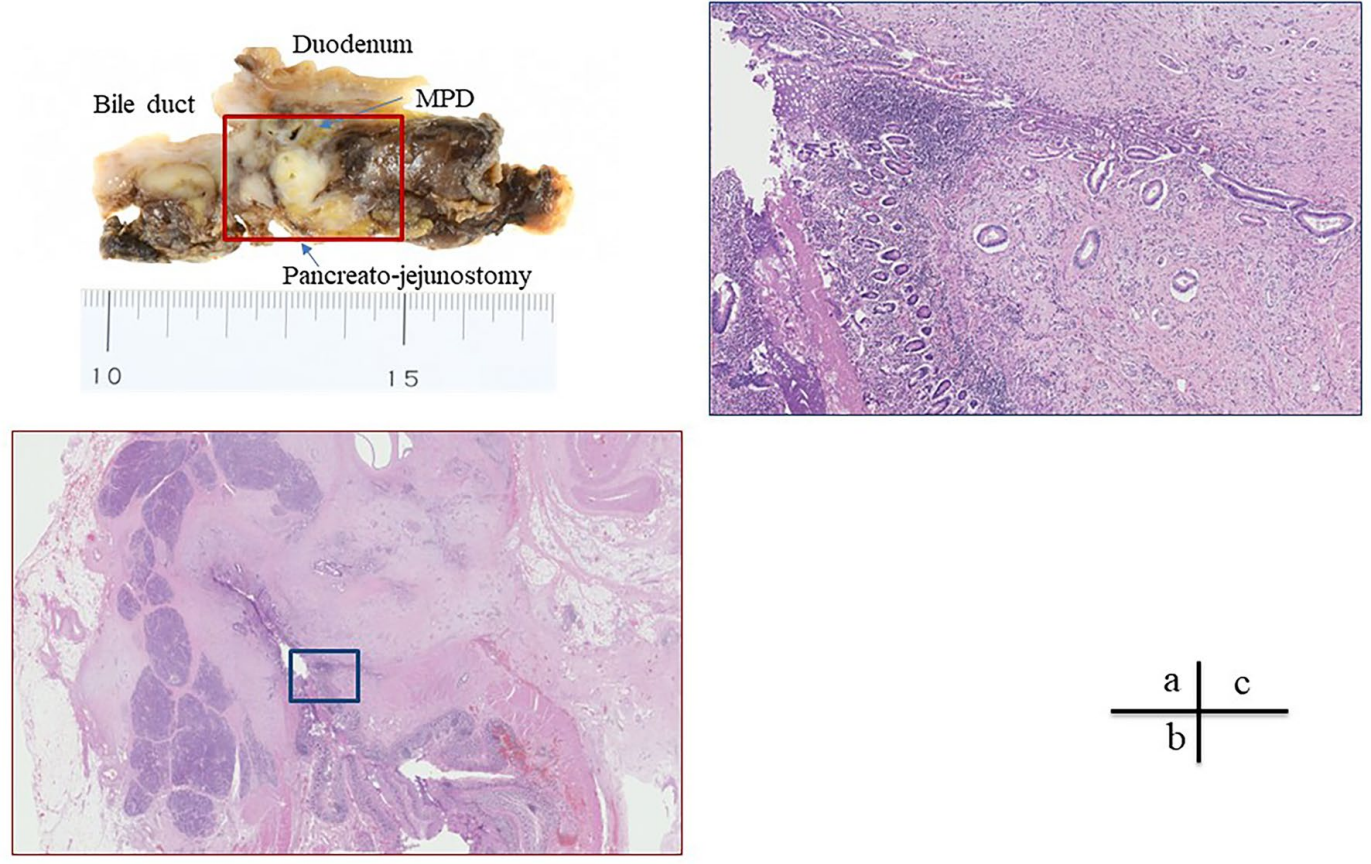
Pathological findings of the tumor. **a** A 3 cm indistinct tumor in the pancreatic head involves to involve the prior pancreaticojejunostomy. **b** The tumor exhibited extensive invasive growth, infiltrating into the anastomosed jejunum, ranging from the muscularis propria to the mucosa. **c** The histological type was moderately to poorly differentiated invasive ductal carcinoma, characterized by ductal formation to acinar patterns and isolated atypical cell proliferation against a background of abundant fibrosis

**Fig. 5:**
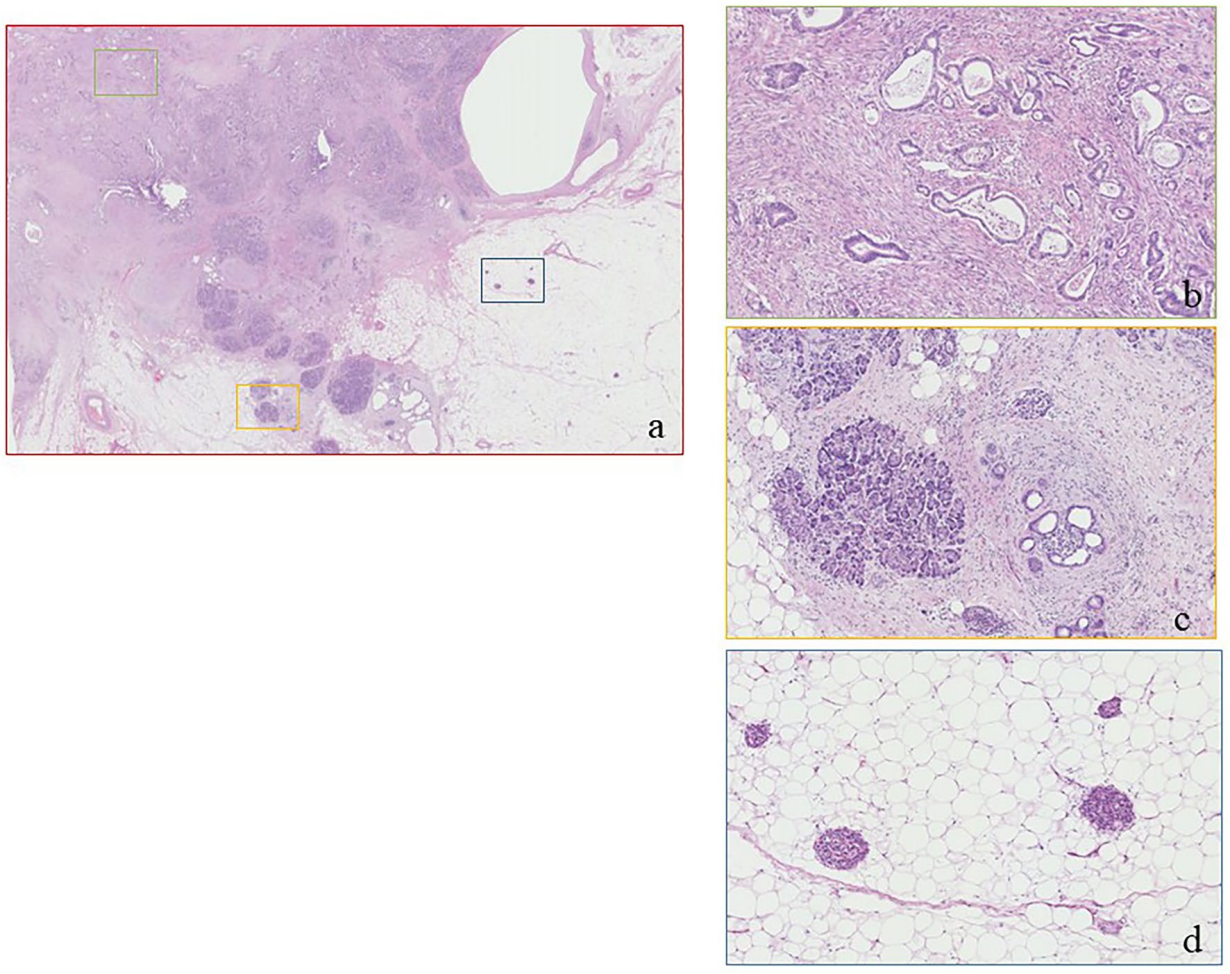
Pathological findings of the non-tumorous pancreatic parenchyma. **a **Significant fibrosis in the non-cancerous portion of the pancreatic head is observed.** b **The acinar cells in these regions are markedly reduced, which is consistent with the findings of advanced chronic pancreatitis.** c **There is no evidence of inflammatory cell infiltration suggestive of acute inflammation in the non-cancerous pancreatic parenchyma. **d **There are scattered islets of Langerhans amidst fatty infiltration of the pancreatic parenchyma, suggesting that the extensive fibrosis is associated with chronic pancreatitis

Macroscopic findings revealed a 3 cm indistinct tumor in the pancreatic head, which appeared to involve the prior pancreaticojejunostomy (Fig. 4a). Microscopically, the tumor corresponded almost exactly with the gross lesion observed. The tumor exhibited extensive invasive growth, infiltrating into the anterior and posterior pancreatic fat tissues, the duodenum, and the anastomosed jejunum, ranging from the muscularis propria to the mucosa (Fig. 4b), as well as into the bile duct wall. The histological type was moderately to poorly differentiated invasive ductal carcinoma, characterized by ductal formation to acinar patterns and isolated atypical cell proliferation against a background of abundant fibrosis (Fig. 4c). The final diagnosis was invasive ductal carcinoma of the pancreas with regional lymph node metastases. The pathological tumor response rate was approximately 40% and the treatment effect was classified as Grade Ib according to Classification of Pancreatic Carcinoma 8th edition by Japan Pancreas Society. Regional lymph node metastases, and R0 resection were confirmed pathologically. Consequently, the tumor was diagnosed as pT2N1M0, Stage IIB, per the TNM eighth edition. The histopathological examination also revealed significant fibrosis in the non-cancerous portion of the pancreatic head (Fig. 5a). The acinar cells in these regions were markedly reduced, which is consistent with the findings of advanced chronic pancreatitis (Fig. 5b). Furthermore, there was no evidence of inflammatory cell infiltration suggestive of acute inflammation in the non-cancerous pancreatic parenchyma (Fig. 5c). In addition, there were scattered islets of Langerhans amidst fatty infiltration of the pancreatic parenchyma, suggesting that the extensive fibrosis was associated with chronic pancreatitis (Fig. 5d).

Postoperative acute cholangitis was occurred possibly due to the afferent limb of previous jejuno-jejunostomy, which was treated by intestinal decompression using jejunostomy tube. The patient recovered well with no signs of recurrence 6 month after surgery.

## Discussion

As reported in a nationwide epidemiological survey conducted in Japan, patients with chronic pancreatitis exhibited a risk of pancreatic cancer that is approximately 11.8 times higher than the general population [11]. Notably, the patient presented in this case had a history of side-to-side pancreaticojejunostomy, a procedure commonly recognized as the Partington procedure, performed 40 years previously. The purpose of such interventions is multifaceted and primarily aimed at alleviating pain and mitigating the risk of further complications, including pancreatic cancer [8, 12]. Ueda et al. reported that the incidence of pancreatic cancer was significantly lower in patients who had undergone surgery for chronic pancreatitis than in those who had not (hazard ratio 0.11; 95% confidence interval, 0.0014–0.80; *p* = 0.03).

Although no pathological signs of acute pancreatitis were observed in this case, recent studies highlight the importance of considering acute pancreatitis as a risk factor for pancreatic adenocarcinoma. Munigala have highlighted that the long-term risk of pancreatic ductal adenocarcinoma is elevated following episodes of acute pancreatitis, irrespective of the cause, underscoring the need for careful monitoring in patients with any form of pancreatitis.

The surgical approach of pancreaticojejunostomy has been proven to prevent the progression of chronic pancreatitis by modulating pancreatic duct drainage, thereby addressing the root cause of inflammation [9]. Zheng et al. emphasized the potential preventative effect of early surgical intervention on the onset of pancreatic cancer and found that the interval between the diagnosis of chronic pancreatitis and surgical intervention was an independent risk factor for developing pancreatic cancer [9]. These findings underscore the possible protective effect of surgical interventions such as the Partington procedure against carcinogenesis, particularly when performed early in the disease course. In this case, the Partington procedure appears to have effectively moderated the chronic inflammation, as evidenced by the diminished pancreatic calcifications observed in the computed tomography conducted prior to the pancreaticoduodenectomy.

The prior pancreaticojejunostomy posed considerable surgical intricacies due to altered anatomy. If the tumor had been distant from the prior pancreaticojejunostomy, surgery to preserve this lateral anastomosis might have been possible. In that case, a new pancreaticojejunostomy could have been avoided by preserving the elevated jejunum. In this case, the previous pancreaticojejunostomy did not extend to the tail of the pancreas, and a portion of the pancreatic tail could be retained. Although the remaining pancreas was small, the patient's glucose tolerance was maintained after surgery, and there were no significant digestive or absorptive disturbances; therefore, we believe there was considerable benefit in preserving the small pancreas.

Regarding pancreatic cancer that develops after surgery for chronic pancreatitis, Zhen reported that patients who underwent surgery for CP showed a higher cumulative incidence of PC with rates of 1.48% at 3 years, 2.63% at 6 years, and 3.71% at 9 years after surgery [9]. However, we could not find any detailed reports on PC post-pancreatitis surgery in the English literature, and there were only two case reports in Japanese. Nagahisa et al. reported a case of intraductal papillary mucinous carcinoma of the pancreatic body that developed 25 years after Partington’s procedure and was treated with distal pancreatectomy [13], and Tamura et al. reported a case of pancreatic invasive ductal carcinoma that developed 30 years after surgery for chronic pancreatitis and was treated with distal pancreatectomy ^15^ (Table 2). Including the present case, in all three instances, PC was detected after more than 25 years following surgery for CP, with tumor sizes exceeding 2.5 cm. It should be noted that pancreatic cancer after surgery for chronic pancreatitis can occur even after a long time after surgery, and physicians need to follow-up with patients with CP over a long period of time, even after surgery for CP.

**Table 2:** Summary of the reported cases

Author	Year	Sex	Age (year)	Surgery for CP	Treatment for PC	Time from CP surgery to pancreatectomy (year)	Tumor size (mm)	Pathology
Nagahisa^9^	2013	Male	76	Partington	DP	25	100	IPMC, T3N0
Tamura^10^	2022	Female	71	Pancreaticojejunostomy	NAC → DP	30	25	Mod, T3N0
Our case	2023	Male	72	Partington	NAC → PDPVR	43	32	Mod, T3N1

*DP* distal pancreatectomy, *IPMC* intraductal papillary mucinous carcinoma, *NAC* neoadjuvant chemotherapy, *Mod* moderately differentiated adenocarcinoma, *PDPVR* pancreaticoduodenectomy with portal vein resection

In conclusion, while surgical interventions for chronic pancreatitis reduce the risk of pancreatic cancer, it should be noted that pancreatic cancer can occur even after a long time after the surgery. Further studies and case series would be invaluable in elaborating best practices for such complex cases.
